# Artificial Intelligence-Enabled Point-of-Care Echocardiography: Bringing Precision Imaging to the Bedside

**DOI:** 10.1007/s11883-025-01316-9

**Published:** 2025-07-07

**Authors:** Sasha-ann East, Yanting Wang, Naveena Yanamala, Kameswari Maganti, Partho P. Sengupta

**Affiliations:** Division of Cardiovascular Diseases and Hypertension, Department of Medicine, Rutgers Robert Wood Johnson Medical School, 125 Paterson St, New Brunswick, NJ 08901 USA

**Keywords:** Artificial intelligence, Point-of-care ultrasound, Deep learning, Generative AI

## Abstract

**Purpose of Review:**

The integration of artificial intelligence (AI) with point-of-care ultrasound (POCUS) is transforming cardiovascular diagnostics by enhancing image acquisition, interpretation, and workflow efficiency. These advancements hold promise in expanding access to cardiovascular imaging in resource-limited settings and enabling early disease detection through screening applications. This review explores the opportunities and challenges of AI-enabled POCUS as it reshapes the landscape of cardiovascular imaging.

**Recent Findings:**

AI-enabled systems can reduce operator dependency, improve image quality, and support clinicians—both novice and experienced—in capturing diagnostically valuable images, ultimately promoting consistency across diverse clinical environments. However, widespread adoption faces significant challenges, including concerns around algorithm generalizability, bias, explainability, clinician trust, and data privacy. Addressing these issues through standardized development, ethical oversight, and clinician-AI collaboration will be critical to safe and effective implementation.

**Summary:**

Looking ahead, emerging innovations—such as autonomous scanning, real-time predictive analytics, tele-ultrasound, and patient-performed imaging—underscore the transformative potential of AI-enabled POCUS in reshaping cardiovascular care and advancing equitable healthcare delivery worldwide.

## Introduction

The integration of artificial intelligence (AI) in clinical medicine is revolutionizing the healthcare industry. It is rapidly reshaping the standards for patient-centered care and medical research. In cardiovascular medicine, AI has influenced our standard for diagnosing disease, delivering treatment, and predicting future risks. The technological advancements in various cardiac imaging modalities make the field ripe for AI integration. Specifically, point-of-care ultrasound (POCUS) has rapidly expanded into various clinical settings due to the improvements in probe size and functionality. The portability of POCUS devices offers real-time imaging results that can facilitate immediate diagnosis and management of many pathologic conditions. Consequently, this could expand the pool of providers using ultrasound guidance for decision-making. However, a significant barrier limiting POCUS use is the need for operator expertise in image acquisition and interpretation, which is critical for accurate and efficient medical decision-making. Artificial intelligence-enabled POCUS has the potential to assist clinicians in the acquisition and interpretation of diagnostic echocardiogram images to mitigate the variability in operator proficiency. This review summarizes the transformative landscape of AI-enabled POCUS. Besides the potential, we discuss the challenges of AI integration in POCUS, including its ethical considerations and potential risks. Finally, we outline the future directions of AI-enabled POCUS to be examined, highlighting pragmatic steps required for innovating bedside diagnostic imaging.

## General Advances in AI for Cardiac Imaging

**Fig. 1:**
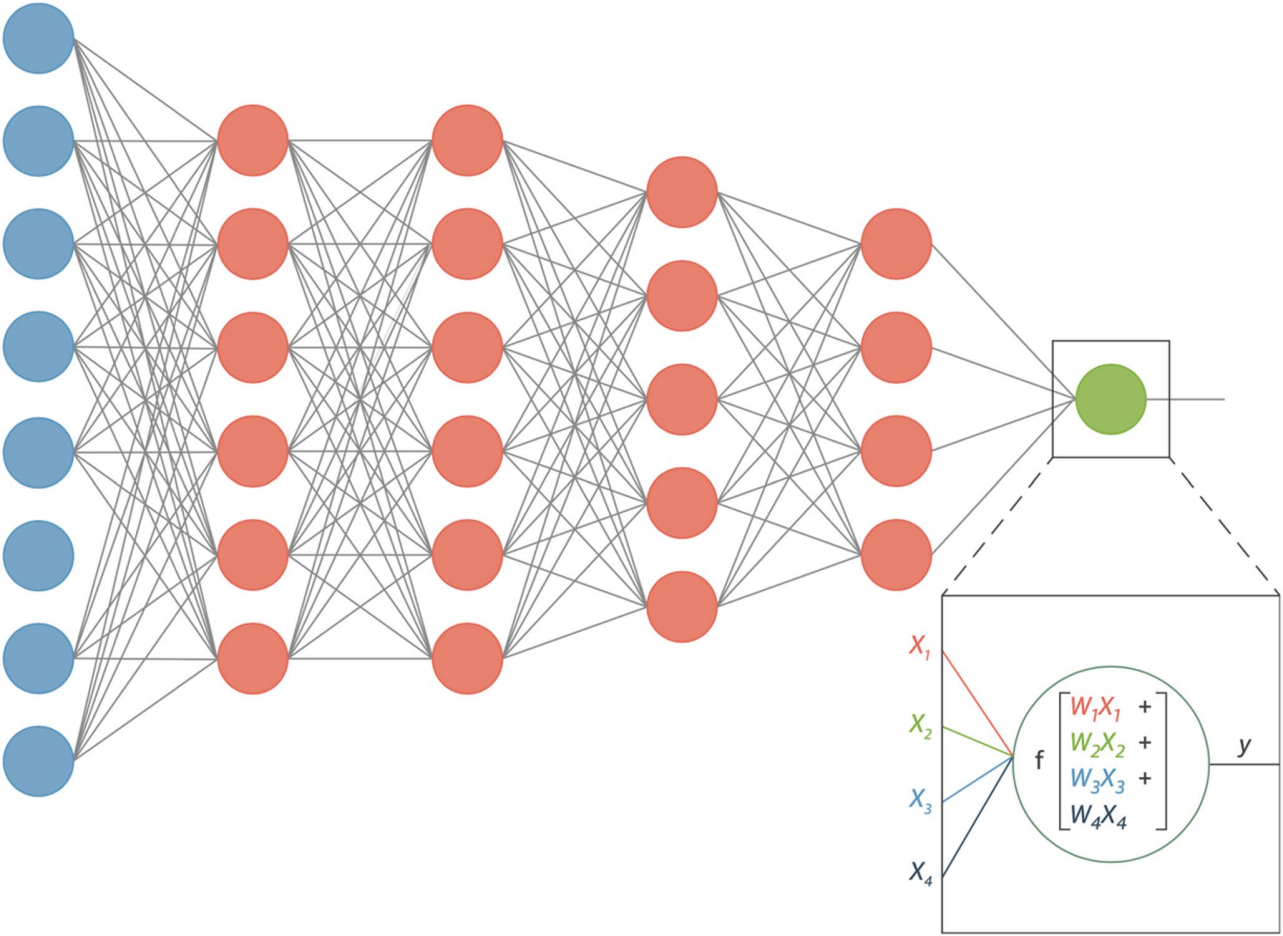
Schematic drawing of an artificial neural network with 5 layers. The input and output layers in this drawing are indicated in blue and green; the hidden layers are in pink. The output node y has been highlighted to show explicitly the mathematical operation that occurs in every neuron. Each input x_i_ to a neuron has its own weight w_i_, indicated here with separate colors. Subsequently, a nonlinear function f is applied to the result. Reproduced from Litjens et al. [1]

**Fig. 2:**
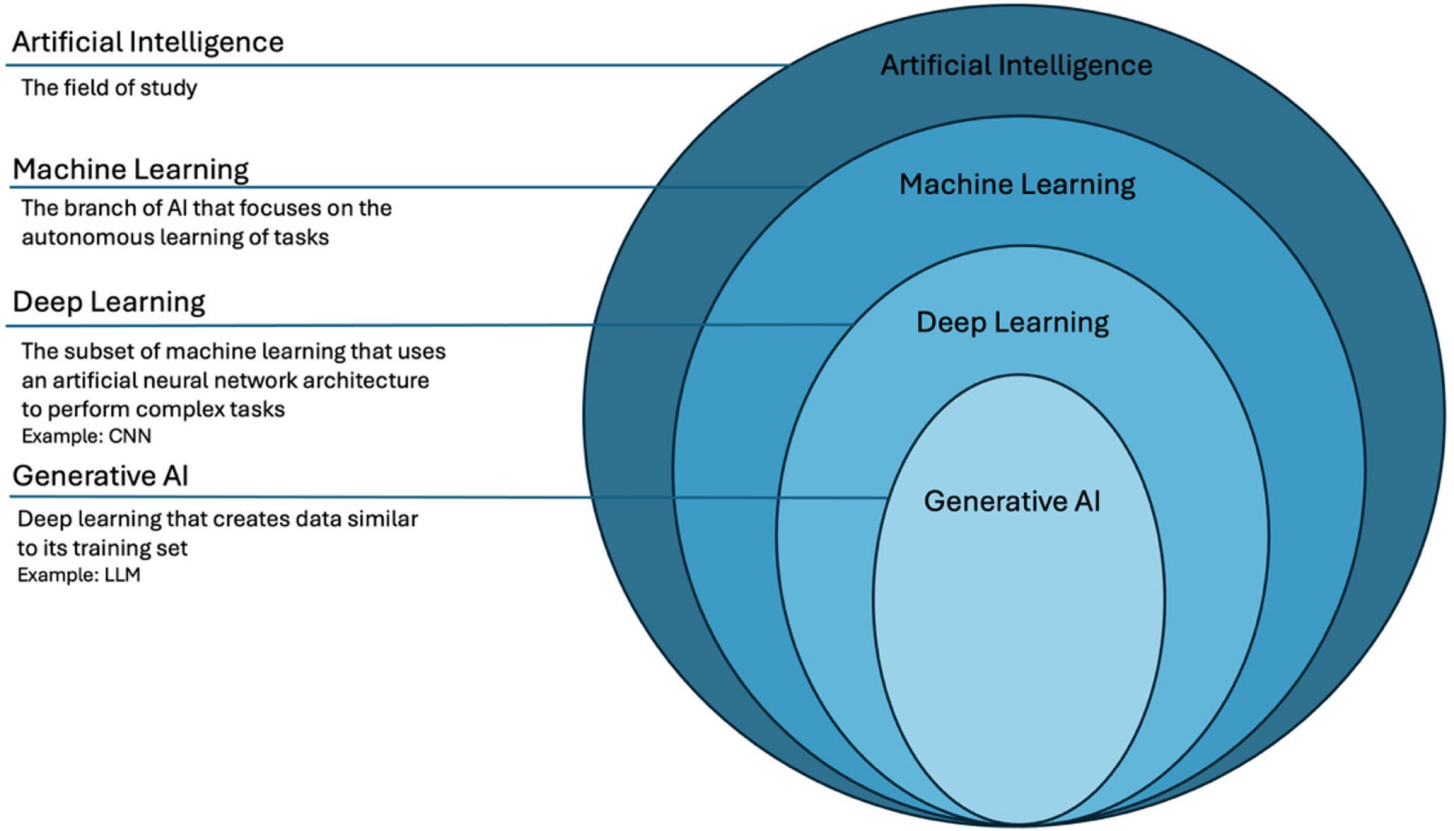
The order of artificial intelligence systems (No citation is necessary. The first author created the graphic in Microsoft PowerPoint.)

The advent of machine learning has allowed for significant advancements in the integration of artificial intelligence and cardiac imaging. The principle of deep learning, which uses neural network architecture to learn from complex datasets autonomously, leverages the massive amounts of data generated from cardiac imaging modalities to automate various tasks and provide deeper insight into disease states, as seen in Fig. 1 [1]. Convolutional Neural Network models are uniquely suited for echocardiography due to their capacity to classify large, high dimensional data such as images and videos [2]. Similarly, generative AI introduces an additional layer of automation to cardiac imaging. Large language models, trained on extensive medical literature and structured reports, can generate coherent and standardized content, potentially streamlining echocardiogram reporting and reducing clinician workload (Fig. 2).

Numerous studies have shown the accuracy and feasibility of AI-guided tasks in CART-based echocardiography. Research has focused on three key areas: image acquisition, image interpretation and diagnostics, and workflow optimization. Proficiency in image acquisition requires time and resources for extensive training and practice. The demand for experienced cardiac sonographers far exceeds the supply of trained personnel. Consequently, regions with limited echocardiogram availability suffer from healthcare disparities that impact patient outcomes. To address these challenges, advancements in AI-assisted image acquisition have aimed to reduce operator dependency and expand access to ultrasonography. Narang et al. investigated a deep learning algorithm trained on ultrasound probe movements to guide probe movement during image acquisition. The nurses who used the DL-guided ultrasound devices could successfully obtain standard echocardiographic images of diagnostic quality despite limited ultrasound training [3]. Similarly, a machine learning algorithm has been developed to assist first-year medical students in acquiring key echocardiographic views—apical 4-chamber, apical 2-chamber, and parasternal long axis—for left ventricular ejection fraction (LVEF) estimation. With real-time feedback provided by the AI-enabled device, students could obtain images of sufficient quality for accurate LVEF assessment [4].

Several key studies also addressed inter-observer variability in echocardiographic measurements [5]. The left ventricular ejection fraction, a defining parameter in echocardiography, conventionally requires manual tracing of the ventricular endocardial borders or, less reliably, visual estimation for quantitative assessment. These methods are prone to diagnostic inaccuracies, which makes it challenging to rely on LVEF to guide management. Several deep learning algorithms have proven to be highly accurate and precise in LVEF measurements. Knackstedt et al. showed the ability of a machine learning algorithm, AutoLV, to generate automated EF measurements with similar accuracy to manual counterparts. Furthermore, there was no variability in the computerized measurements, and the images were analyzed in about 8 seconds [6]. In another study, researchers developed and validated an AI model that could automatically classify, segment, and annotate echocardiograms and Doppler measurements with high accuracy [7].

Deep learning algorithms have also been applied to highly complex challenges, including disease detection. In stress echocardiography, Upton et al. developed a DL algorithm for automated detection of features on stress echocardiogram images that correspond with severe coronary artery disease on invasive coronary angiography. The model achieved high accuracy compared to experienced echocardiogram readers [8]. In another study, a DL algorithm was trained to identify subtle differences in cardiac wall motion abnormalities related to acute myocardial infarction versus stress cardiomyopathy. The deep learning model outperformed cardiologists in identifying the two disease states on echocardiographic images [9].

Lastly, optimizing sonographer workflow has been another key area of research. Delays in echocardiography often stem from poor image quality, as non-diagnostic studies frequently require additional imaging. Without significant investment in ongoing sonographer education, real-time feedback on image quality remains limited. A deep learning model has been developed to autonomously evaluate image quality based on end-systolic echo data, with automated scores demonstrating a strong correlation with manual assessments by expert cardiologists [10]. Integrating this real-time feedback system could reduce operator variability, minimize non-diagnostic echocardiograms, and ultimately improve efficiency in patient care.

## AI Integration with Point-of-Care Ultrasound: Current Applications

Point-of-care ultrasound is moving to the forefront of clinical practice due to its versatility and convenience. The ability to perform rapid multi-organ assessment at the bedside significantly improves workflow and decreases delays in care. Common uses for POCUS include the identification of pulmonary edema, internal hemorrhage, and peripheral veins for insertion of central venous catheters. For the cardiovascular system, focused cardiac ultrasound (FCU) equips clinicians with a powerful tool for cardiac evaluation, improving their ability to make informed clinical decisions. Emergency medicine, trauma, critical care, and cardiology providers use FCU to enhance their physical exam and to make critical time-sensitive diagnoses. FCU doesn’t require extensive training compared to comprehensive echocardiography, as it focuses on obtaining the most relevant images to confirm or rule out cardiac abnormalities. Despite the limited images, FCU can provide reasonably accurate assessments of chamber size, ventricular function, pericardial effusion, and central venous volume. This facilitates patient triage and workflow, and can better refine downstream testing. FCU can also aid in managing cardiac arrest, allowing for a more rapid assessment of reversible causes [11]. Still, there are significant barriers to widespread adoption. FCU does not replace comprehensive echocardiography due to its limited scope. It may be suboptimal for evaluating more complex cardiac pathology such as valvular disease, diastolic dysfunction, or pulmonary hypertension.

Furthermore, apical views are more challenging to obtain for an accurate interpretation of wall motion abnormalities. FCU requires investment in formal training with competency assessments and ongoing maintenance of skills. Integrating artificial intelligence with POCUS can move cardiac ultrasound beyond the echo lab, improving access to efficient, high-quality care.

### Image Acquisition and Quality Improvement

**Fig. 3:**
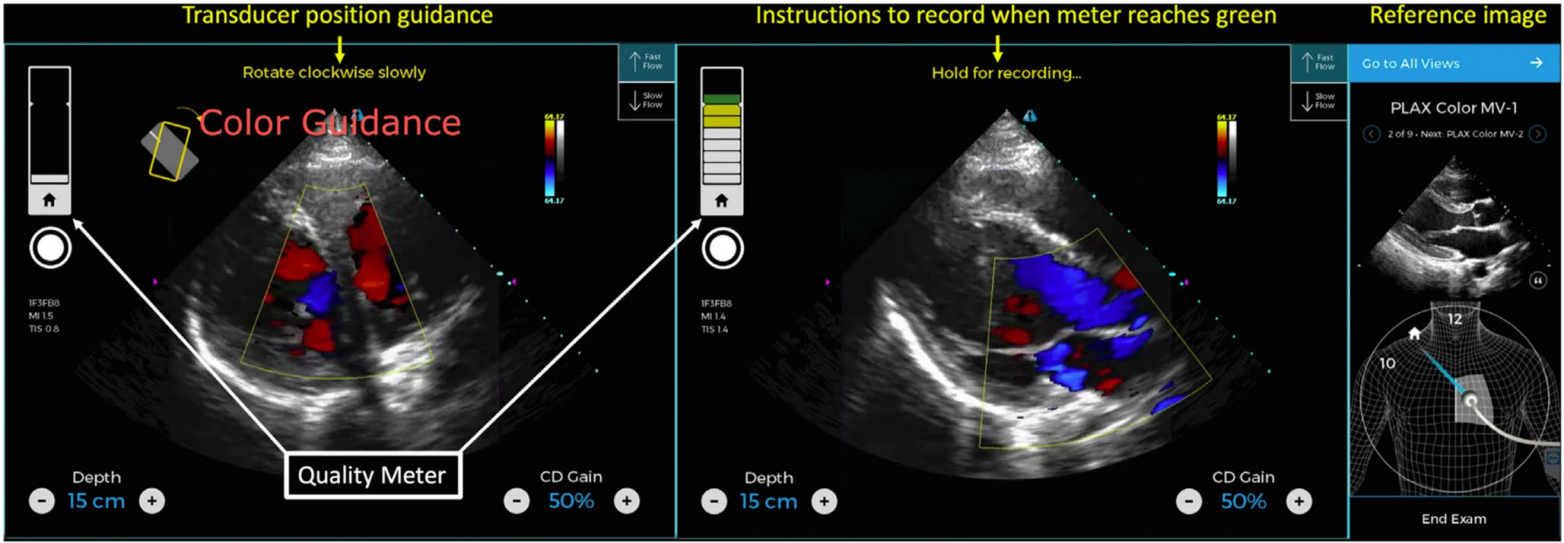
AI guidance user interface. Initial (*left*) and captured (*center*) PLAX color Doppler image of aortic outflow and mitral regurgitation using AI guidance. The reference image (*right*) instructs the novice where to place the transducer. Instructions for transducer movement result in an optimal image and an increase in the quality of the meter from white to yellow to green. Once the meter is in green, the scanner is instructed to manually acquire the image. Adapted from Peck et al. [14]

POCUS has become widely accepted among various providers, enhancing patient access to care. However, the adoption rate has outpaced formal POCUS training, which could lead to potential patient harm. Hence, there is a need for training and standardization protocols to reduce the risks of inappropriate use and misinterpretation by those with less experience. A survey of hospitals showed that only a fraction of providers that use POCUS felt confident in their acquisition and interpretation skills. Furthermore, knowledge deficits regarding POCUS’s limitations and appropriate use were highlighted [12]. AI has the potential to bridge that gap by leveraging deep learning techniques to aid image acquisition. Algorithms have been trained to guide probe positioning and provide immediate feedback on image quality, helping users adjust their scanning technique in real time. In one study, AI-enabled devices significantly improved scan times and image quality scores among novice users [13]. In another study, AI-guided acquisition showed promise in disease-specific screening. Peck et al. demonstrated the feasibility of AI-guided POCUS for rheumatic heart disease detection. With only one day of training, users were able to acquire diagnostic-quality images in over 90% of cases as seen in Fig. 3 [14]. These results suggest that AI-enhanced ultrasound can be a powerful adjunct to traditional training pathways, improving access and consistency in image acquisition.

### Image Interpretation and Diagnostics

**Fig. 4:**
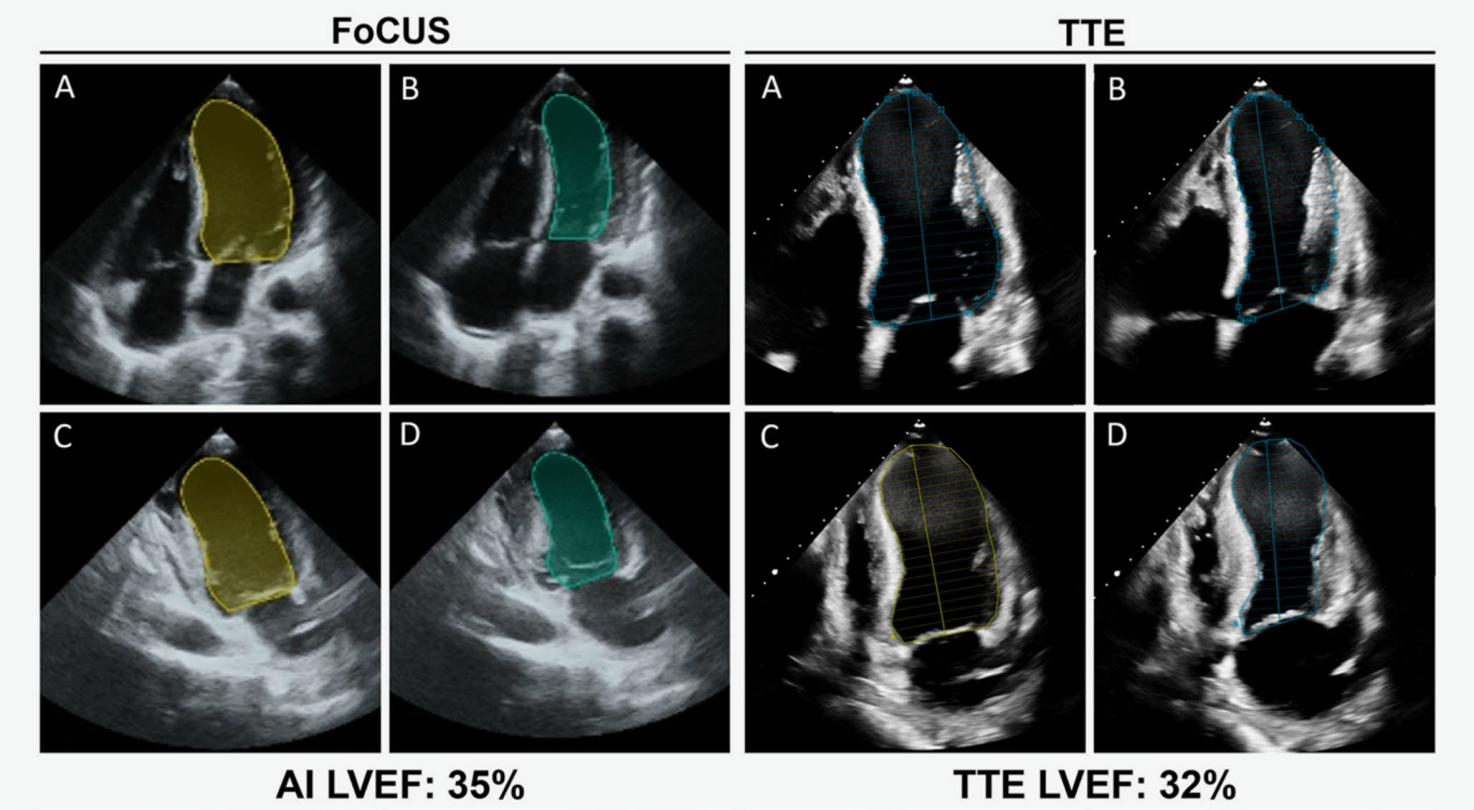
AI-assisted LVEF FoCUS and TTE image acquisition. LV cavity tracings by automated AI and manually by an echocardiographer of the A4C end-diastolic (**A**) and -systolic (**B**) frames and A2C end-diastolic (**C**) and -systolic (**D**) frames to calculate LVEF. Reproduced from Motazedian et al. [15]

Automated interpretation could be particularly beneficial in POCUS. AI could ensure rapid and accurate determination of various POCUS parameters such as ventricular systolic function, pericardial effusion size, and volume status. These parameters are susceptible to diagnostic inaccuracies, especially with limited views. Machine-learning algorithms designed to provide automated POCUS image interpretation have been promising as seen in Fig. 4 [15]. Asch et al. tested the accuracy of fully automated ML-enabled POCUS devices for quantification of LVEF. The study noted that the POCUS-derived automated LVEF measurements were comparable to reference echocardiograms interpreted by expert cardiologists. The accuracy was even sustained when single views were analyzed individually. The study also showed that automated LVEF assessments on POCUS images obtained by novices were almost as accurate as reference measurements [16]. Similarly, deep learning algorithms for object detection have been developed to identify pericardial effusions with high sensitivity and specificity. One such tool processed images in just 57 milliseconds—an essential advantage for this time-sensitive and potentially life-threatening diagnosis [17].

Advancing automated detection of cardiovascular diseases is a key focus of AI research. Convolutional neural networks can uncover subtle patterns in echocardiographic data that may elude human perception, enabling the development of screening tools for subclinical disease detection. Hathaway et al. developed a machine learning model using POCUS data that identified textural features for left ventricular remodeling and dysfunction. The model predicted the presence of LV remodeling with good accuracy, and its predictions were strongly associated with significant adverse cardiac events [18]. In another study, Oikonomou et al. trained an algorithm using POCUS images to distinguish amyloid (ATTR) and hypertrophic cardiomyopathies from controls with high accuracy. Notably, among patients who received a POCUS exam before their diagnosis, the median time between the AI-designated abnormalities and confirmatory testing was approximately 2 years. The study also found that patients without known cardiomyopathy but exhibiting a high-risk AI-derived phenotype had an increased risk of mortality. These findings highlight the potential of AI-enhanced POCUS as a screening tool for high-risk populations [19].

## Challenges and Limitations of AI in POCUS

### Technological Challenges and Ethical Considerations

**Fig. 5:**
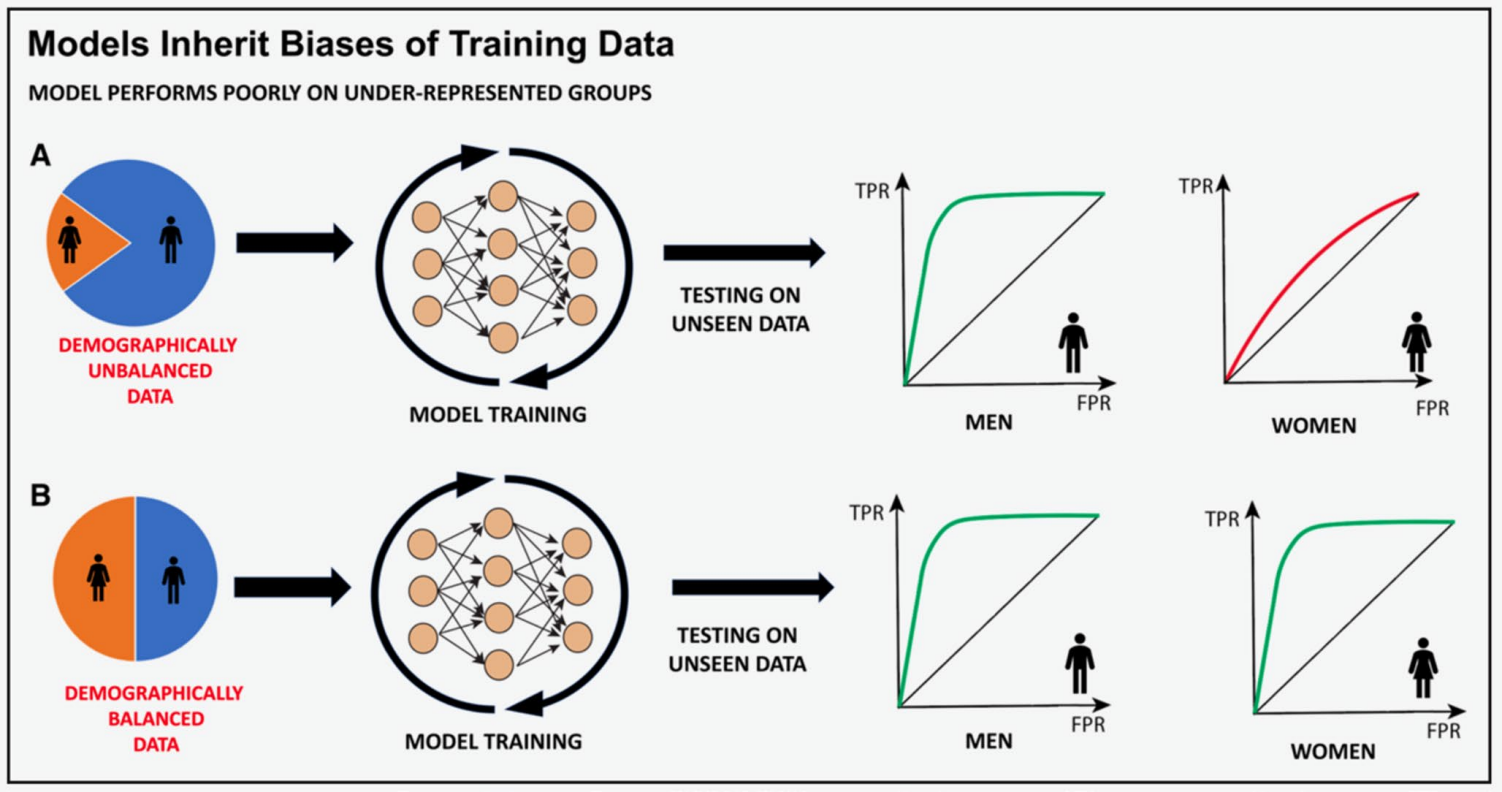
Imbalanced training data can lead to biased models. Although it is unclear whether any under representation in training data or under-representation relative to the population portion of a demographic group is a driver, imbalanced training data sets (as shown in model A) have led to models that perform worse for select population sub groups compared with models trained where demographic groups are better balance (model B). For this reason, investigating bias in model predictions and imbalance in training data is key to creating equitable models. FPR indicates false positive rate; and TPR, true positive rate. Reproduced from Vrudhula et al. [21]

While the integration of artificial intelligence can bring significant improvements to medical imaging, technical challenges and ethical considerations require ongoing appraisal and intervention. Deep learning models often underperform in real-world settings compared to the training environment due to a lack of generalizability [20]. Successful DL algorithms depend on broad exposure to variations in image quality, patient demographics, and operator skill, which requires a robust training dataset. ML models are very sensitive and can perpetuate biases within skewed data as seen in Fig. 5 [21]. Disparities in age, sex, gender, geographic, and socioeconomic status within training data can lead to biased predictions, resulting in incorrect diagnoses and missed opportunities for appropriate treatment [21]. Medical acuity can also introduce bias, especially when training on imaging data that represent the extremes of a disease spectrum. Such data may not be generalizable to mild or moderate forms of the disease. Errors in labeling training data can also lead to inaccuracies, as small measurement mistakes may cause significant misclassifications. Ultimately, the lack of standardization in developing AI algorithms makes them prone to bias. Ongoing research is aimed at standardizing algorithm development and mitigating bias in every step of training. For imaging-centered models, it has been shown that including non-imaging factors such as clinical and demographic data can improve model generalizability [22]. Additionally, generative AI can create synthetic training data sets with balanced patient demographics. Even after model training is complete, routine reporting of model performance should be instituted to help maintain model transparency and provide up-to-date guidance on how the results should be applied [21].

### Clinical Integration and User Acceptance

Successful clinical integration of AI-generated data depends on clinician confidence in AI algorithms. However, the highly complex nature of neural networks often makes it difficult for users to understand the reasoning behind a model’s conclusion, which can hinder adoption in clinical decision-making [21]. There is considerable effort in making AI explainable to enhance model transparency, build trust, and support informed decision-making [23]. To bridge the gap between AI development and clinical application, data scientists and clinicians must collaborate at every model development step. By incorporating clinician input early on, AI models can better target real-world clinical needs and improve usability [24].

Healthcare systems must also ensure that the clinician remains knowledgeable in using and appraising AI-generated data. This requires an infrastructure for quality assurance and maintenance of competency. Baum et al. observed that trainees exposed to AI-enabled POCUS did not report increased trust in the AI features or a perceived increase in scanning competence despite their improved performance compared to standard POCUS users [13]. Conversely, there is concern regarding the potential for over-reliance on AI, known as automation bias, which could lead to using AI as a substitute for clinical judgment. There must be a sufficient understanding of the strengths and weaknesses of the dataset used to train an ML algorithm before applying its prediction to an individual patient [21]. Otherwise, over-reliance on an algorithm trained on skewed or insufficient data may have a negative impact on patients.

### Data Management and Patient Privacy

Artificial intelligence algorithms require a vast amount of patient data for training, posing a risk to patient privacy. Rigorous safety and compliance measures need to be in place to ensure data security. Proper data preparation includes de-identifying all images and the Digital Imaging and Communications in Medicine metadata. This ensures patient privacy if datasets are posted to open-source research platforms. Similarly, data storage efforts must prioritize data safety while keeping the data accessible across institutions. Cloud-based storage solutions offer a relatively secure way of storing and sharing data [25].

## Future Directions and Innovations

**Fig. 6:**
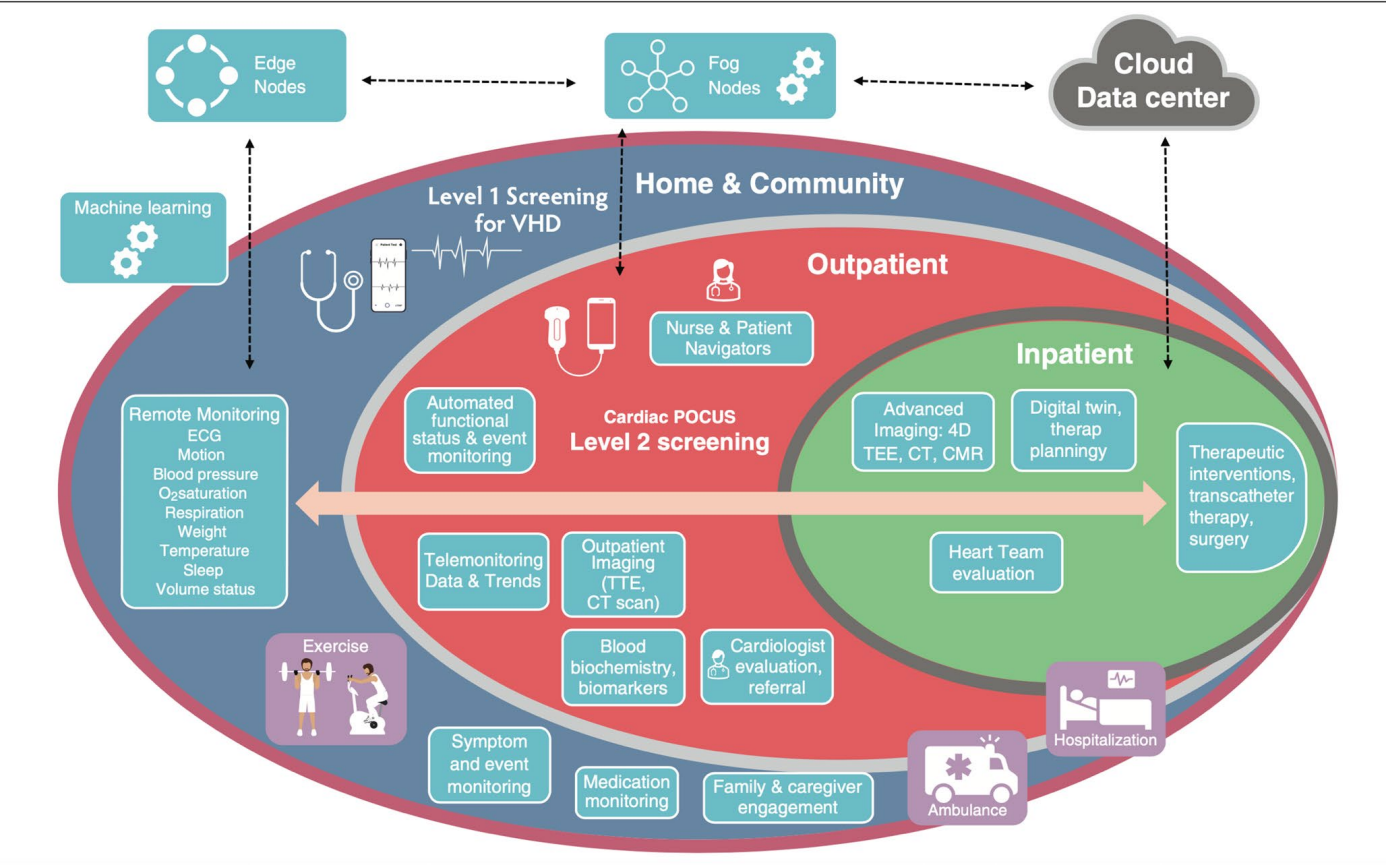
Digital innovations in care delivery for valvular heart disease. The schematic conceptualizes machine learning techniques using ECG, wearable devices, and physiological sensor-based data for screening, individualized care coordination, and follow-up strategies. Screening in communities can be triggered with the use of wearables, devices, and remote patient monitoring systems. The appropriate triggers can lead to specialized consultations, with additional screening (using cardiac POCUS imaging techniques) and optimization of downstream testing, evaluation, and timing of interventions. ECG = electrocardiogram. VHD = valvular heart disease. POCUS = point-of-care ultrasound. TTE = transthoracic echocardiogram. TEE = transesophageal echocardiogram. Adapted from Sengupta et al. [28]

The evolving synergy between AI and emerging technologies is reshaping the landscape of POCUS education and outpatient diagnostics. Integrating artificial intelligence with virtual reality, for example, can enhance POCUS education and facilitate the maintenance of competency. The immersive, simulated environments can allow learners to practice procedural skills without risk to patient safety [26]. As POCUS becomes more widely adopted in outpatient settings, primary care physicians may increasingly rely on it to augment the physical exam, guide clinical decisions, and reduce unnecessary testing. Automated structure recognition and image interpretation can further streamline workflow and reduce the likelihood of missed incidental findings. On another note, tele-ultrasound, an emerging form of telemedicine that enables remote expert guidance and interpretation, will continue expanding its footprint. Studies have demonstrated its effectiveness in image acquisition, cost-effective screening, and clinical management [27]. Artificial intelligence can bolster this initiative by facilitating ultrasound education and image acquisition to help improve diagnostic accuracy. Beyond ultrasound, AI is also being integrated into other cardiac assessment tools, such as electronic stethoscopes and electrocardiograms. Combined, these AI-augmented technologies could create a robust and accessible suite of screening tools for frontline healthcare providers as seen in Fig. 6 [28].

One of the most promising frontiers for AI-enabled POCUS is personal-use ultrasound, where patients self-acquire images and transmit them to their healthcare provider. This approach has shown promise in a study training hemodialysis patients to perform lung ultrasounds using AI-enabled POCUS devices. With integrated B-line counting capabilities, patients could capture images of sufficient diagnostic quality for the detection of pulmonary congestion [29, 30]. These findings lay the groundwork for integrating AI, self-performed ultrasound, and telemedicine - an endeavor that can have far-reaching global health benefits.

Lastly, another promising direction is the integration of AI with robotic ultrasound systems.

Robotic ultrasound has already demonstrated feasibility in obtaining diagnostic-quality images for remote interpretation. In one study, the use of robotic ultrasound with teleconsultation significantly reduced the time to diagnosis compared to traditional workflows, and patients rated the experience as equivalent or superior to standard care [31]. While research on robotic cardiac ultrasonography remains limited, recent work in thyroid imaging has shown that an autonomous robotic scanning system can successfully acquire and interpret images using deep learning algorithms. This framework offers a potential foundation for developing AI-driven robotic POCUS applications in cardiac imaging [32].

## Conclusion

The integration of artificial intelligence with point-of-care ultrasound (POCUS) is reshaping the future landscape of diagnostic imaging and clinical practice. Artificial intelligence technologies can enhance the accuracy and efficiency of POCUS imaging by addressing key challenges like operator dependency, inter-observer variability, and workflow optimization, facilitating a more streamlined and reliable approach to patient care.

Responsible integration of the two disciplines will require careful appraisal of the technological limitations, ethical considerations, and patient privacy concerns to ensure transparency and equity at every implementation step. The expansion of AI applications in POCUS holds great promise for advancing personalized and global healthcare delivery, offering unprecedented improvements in diagnostic efficiency and patient empowerment.

## Data Availability

No datasets were generated or analysed during the current study.

## Key References

1. • Peck D, Rwebembera J, Nakagaayi D, et al. The Use of Artificial Intelligence Guidance for Rheumatic Heart Disease Screening by Novices. J Am Soc Echocardiogr.2023;36(7):724–732. 10.1016/j.echo.2023.03.001. This novel study demonstrated the feasibility of AI-guided POCUS screening of rheumatic heart disease by non-experts.
2. • Hathaway QA, Yanamala N, Siva NK, et al. Ultrasonic Texture Features for Assessing Cardiac Remodeling and Dysfunction. J Am Coll Cardiol. 2022;80:2187–2201. 10.1016/j.jacc.2022.09.036. This study developed a cardiac ultrasomic-based machine learning model that could predict left ventricular remodeling and major adverse cardiac events.
3. • Vrudhula A, Kwan AC, Ouyang D, Cheng S. Machine Learning and Bias in Medical Imaging: Opportunities and Challenges. Circ Cardiovasc Imaging. 2024;17:e015495. 10.1161/CIRCIMAGING.123.015495. This review explores bias in medical imaging and its potential to influence machine learning models.
